# The use of a standard-length conical tapered stem in hip revision arthroplasty to address Paprosky type I–II femoral defects: a prospective study of 87 patients

**DOI:** 10.1007/s00402-023-04797-y

**Published:** 2023-02-20

**Authors:** Matteo Innocenti, Katrijn Smulders, Mattia Andreotti, Jore H. Willems, Gijs Van Hellemondt, Marc W. Nijhof

**Affiliations:** 1 Department of Orthopaedic Surgery, University of Florence, Florence, Italy; 2 Department of Research, Sint Maartenskliniek, Nijmegen, The Netherlands; 3 Department of Orthopaedic, Ospedale Riuniti Padova, Padua, Italy; 4 Department of Orthopaedic Surgery, Sint Maartenskliniek, Nijmegen, The Netherlands

**Keywords:** Revision THA, Paprosky femoral bone defects, Wagner cone, Conical tapered stem, Subsidence

## Abstract

**Introduction:**

Low-grade femoral defects in revision total hip arthroplasty (rTHA) might be preferably treated with a primary implant. Almost no previous study reported the use of standard-length conical tapered (SLCT) stems in these cases. We analyzed a series of cases using a SLCT stem in rTHA with Paprosky type I–II femoral defects. The purpose of the study was to determine clinical and radiographic outcomes in this series of rTHA.

**Materials and methods:**

We prospectively followed 87 patients undergoing a femoral component rTHA: 53 Paprosky type I and 34 type II femoral defects. Patient-reported measures (Oxford Hip Score, EQ-5D, VAS pain during rest and activity) were administered at baseline, 1 and 2 years post-operatively. Radiographic subsidence overtime was scored. Kaplan–Meier curves were used to evaluate the subsidence over time, the complication-free survival, and the implant survivorship with reoperation and stem revision as endpoints.

**Results:**

The mean follow-up was 72.5 (SD ± 23.9) months. All PROMs significatively improved over time. The average subsidence was 2.8 (SD ± 3.2), 3.6 (SD ± 4.4), and 4.0 (SD ± 4.9) mm at 4, 12, and 24 months respectively. 6 stems had subsidence > 10 mm. The survival without complication was 0.85 (95% CI 0.94–0.77), while the implant survival without reoperation was 0.83 (95% CI 0.95–0.72). The overall stem survival rate was 93.7% (95% CI 0.91–0.97) at 2 years.

**Conclusion:**

The use of a SLCT stem in rTHA with Paprosky type I–II femoral defects demonstrated good survival with low subsidence rates during the first 2 years after surgery. Surgeons should consider the use of this primary prosthesis as a potential treatment during stem revision in cases with limited femoral bone loss.

**Supplementary Information:**

The online version contains supplementary material available at 10.1007/s00402-023-04797-y.

## Introduction

During the past decade, total hip arthroplasty (THA) has become an increasingly common surgical procedure among younger and active patients with a high healthy life expectancy[1–4]. Consequently, there has been an increasing demand for revision THA (rTHA) that may also dramatically increase the re-revision THA burden [5]. Indeed, the risk of subsequent re-revision after a first rTHA has been estimated to be about six times higher than a first revision after a primary THA [6].

Femoral bone loss is one of the main concerns and can be a considerable challenge in the revision total hip arthroplasty. In order to save the diaphyseal bone stock, it would be advantageous to use a primary stem for revision surgery whenever possible [7–9]. Whereas the use of a long revision stem seems to be mandatory to bypass a gross Paprosky type 3 femoral defect providing distal solid fixation [10–16], lower-grade femoral defects might be reasonably managed with a primary implant. The major benefit of using a primary stem in the revision setting is to load the proximal bone stock and preserve meta-diaphyseal bone. Primary stems are surgically easier to implant and show fewer intraoperative complications when compared to longer revision stems [17–19].

We introduced the use of a standard-length conical tapered (SLCT) stem in the management of Paprosky type I–II femoral defects during revision hip arthroplasty. This stem design theoretically achieves stable fixation in every plane mainly by press-fit at the metaphyseal–diaphyseal junction through a long, continuous taper configuration, being particularly useful in the presence of sclerotic bone or after cement removal. The SLCT provides adequate fixation without using the isthmus, and the conical shape offers the freedom of choosing the appropriate femur version regardless of the anatomy of the proximal femur itself. This could be particularly useful in the setting of an isolated femoral revision stem to achieve the properly combined anteversion with an already previously well-fixed acetabular component in place.

To date, excellent results of this type of stem have been reported only in primary THA for patients with abnormal proximal femoral anatomy [20–24].

The purpose of our study was to determine short- and mid-term clinical, radiographic, and functional outcomes in this specific series of rTHA patients.

## Materials and methods

### Participants

This is a registry-based cohort study with a follow-up performed in our specialized high-volume orthopedic Institute. In our revision hip registry, all patients scheduled for rTHA surgery between March 2013 to December 2016 were included. rTHA was defined as the exchange of the cup and/or stem.

For the current study, we selected all patients with a minimum of 2-year follow-up and who received a primary standard-length, grit blasted, titanium monobloc, femoral prosthesis with conical shape and longitudinal splines for initial rotational stability (Wagner Cone, Zimmer, Warsaw IN, United States). The Zimmer Wagner Cone stem has a minimum stem length of 115 mm with a diameter of 13 mm to a maximum length of 127.6 mm with a diameter of 24 mm. The diameter and the length increase accordingly with a fixed 5° taper. The stem is available with a caput-collum-diaphyseal (CCD) angle of 135 or 125 degrees.

The Paprosky classification of bone defects was used to record the amount of femoral bone loss and plan the surgery [11, 25, 26]. Only patients with a Paprosky type I–II bone defect along with no trabecular bone left (sclerotic bone) at the meta-diaphyseal junction were selected. Patients with femoral Paprosky type III bone defects were excluded (n = 3). This resulted in the inclusion of 87 rTHA’s in 87 patients.

### Demographic and clinical assessment

Demographic and surgical characteristics such as age, sex, BMI, and operation side were recorded preoperatively (Table 1). Regular follow-up visits took place at 6 and 12 weeks, and 1 and 2 years post-operatively. Complications were registered at each follow-up and categorized into infection, dislocation, aseptic loosening, periprosthetic fracture, nerve palsy, and any other complication requiring surgery (e.g., prolonged wound drainage, failure of osteosynthesis material) or other medical treatment (e.g., thromboembolic event, urinary tract infections). Despite any kind of complication being recorded, for the purpose of this article, we reported only those complications potentially related to the stem (periprosthetic fractures, dislocation, aseptic loosening, etc.). Patient-reported outcome measures (PROMs) were administered at baseline (preoperatively) and 1 and 2 years post-operatively. PROMs included Oxford Hip Score (OHS) [27], VAS for pain during rest and during activity (range 0–100) [28], and the EuroQol 5 Dimension (EQ-5D-3L) score [29] (Table 1).

**Table 1:** General patient and surgical characteristics and baseline values of outcome measures

Variable	Value
Age at time of surgery (years)^a^	64 ± 12
Sex (male; female) (number of patients)	45; 42
Side (left; right) (number of patients)	41; 46
BMI^a^	29 ± 4
Baseline values outcome measures^a^	
OHS (points)	44.5 ± 12.3
EQ-5D score (points)	0.45 ± 0.3
VAS pain during rest (points)	35.5 ± 29
VAS pain during activity (points)	65.9 ± 27

*BMI* body mass index; *ASA* American Society of Anesthesiologists Classification; *OHS* Oxford Hip Score; *EQ-5D* EuroQol 5 Dimension; *VAS* Visual Analogue Scale

^a^Values are expressed as mean ± SD

### Clinical and surgical procedure

All patients were enrolled for surgery following a standard pre-operative workup according to our hip reconstruction unit protocol with a pelvic X-ray, CT scan, and laboratory tests. If there was any suspicion of infection, an aspiration was added to the workup; a proven prosthetic joint infection was treated accordingly and planned for either a 1- or 2-stage rTHA. An estimation of femoral bone defect was done preoperatively with the use of an X-ray and CT scan, but the definitive amount of bone loss and its relative Paprosky type was recorded intra-operatively after the previous stem extraction. A double pre-operative digital templating, based on calibrated X-rays with a 30 mm metal ball at the level of hips, with both the SLCT stem and a modular or monobloc longer revision stem was performed for every case (Orthoview, Materialise, Leuven, Belgium). Templating provided an estimate of the stem size required for maximal bony contact (equal to the inner diameter of the medullary femoral canal) as well as the appropriate CCD angle and seating needed to correct any pre-existing limb length discrepancy. The final decision on whether to implant an SLCT stem or a modular/monobloc revision long stem was made by the surgeon intra-operatively based on the (rotational) stability of the SLCT stem trial.

Surgical details of both previous and revision surgeries are reported in Table 2 and supplementary material (Table 2.2 in supplementary material).

**Table 2:** Surgical details (previous and revision surgery)

Variable	Value
Type of revision (number, %)	
Full revision	35 (40.9%)
Femoral component only	52 (59.1%)
One stage	64 (72.7%)
Two stage	23 (27.3%)
Previous surgical approach (number, %)	
Direct anterior	1 (1.2%)
Antero-lateral	7 (7.9%)
Direct lateral	14 (15.9%)
Postero-lateral	65 (75%)
Previous stem fixation method (number, %)	
Cemented	3 (3.4%)
Uncemented	84 (96.6%)
Reason for revision (number, %)	
Aseptic loosening	42 (48.3%)
Septic loosening	26 (30%)
Instability/dislocation	13 (14.9%)
Polyethylene or Metal wear	5 (5.7%)
Periprosthetic fracture	1 (1.1%)

Each surgery was performed with a posterolateral approach. Except for cases of infection (in which antibiotic treatment was tailored patient by patient), prophylactic antibiotics were given 15–60 min before incision (2 g of cefazolin). In patients with a BMI ≥ 35 kg/m^2^ the dosage of antibiotics was adjusted to their weight. In the case of a cephalosporin allergy, one gram of vancomycin was administered. During each revision surgery, 6 tissue cultures were taken for microbiological evaluation. Antibiotic prophylaxis was then continued intravenously in the post-operative period until temporary results of intraoperative cultures resulted negative. In the case of (unexpectedly) ≥ 2 positive cultures for the same micro-organism, antibiotics were continued for at least 3 months and the indication for revision was defined as infection.

Weight-bearing, as tolerated, was prescribed. All patients received thromboembolic prophylaxis (subcutaneous enoxaparin 4000 UI/day) for at least 4 weeks following surgery.

### Radiographic assessments

The radiologic evaluation was done using a standardized calibrated antero-posterior pelvic X-ray to evaluate the amount of subsidence over time. We also recorded the presence of any eventual prosthetic and/or periprosthetic fractures or other abnormalities (e.g., radiolucencies, periprosthetic ossifications). Radiographic criteria outlined by Engh et al. [30] were used to evaluate stem subsidence. Femoral stem height in the proximal femoral bone was evaluated by drawing a vertical line along the lateral longitudinal axis of the stem, followed by an additional 2 perpendicular lines drawn horizontally to the first line, the first going through the superior tip of the greater trochanter and the second through the shoulder of the stem [23]. To check the subsidence, the change in trochanter-stem shoulder distance was determined. We used a cut-off of > 10 mm change to define clinically relevant subsidence [31]. In case of subsidence > 10 mm, a CT scan was made to exclude gross outliers in the stem version and any fractures not detected on the regular X-ray.

Radiographic imaging measurements were independently performed by 2 trained orthopedic residents supervised by an experienced orthopaedic surgeon and a radiologist specialized in musculoskeletal radiographs. The operators were not blinded (i.e., which patient was on the X-ray) during these measurements.

Failure was defined as radiographic evidence of loosening or revision of the femoral component for any cause (including osteolysis, instability, component malalignment, infection, etc.).

### Data analysis

Statistical analysis was performed using RStudio (V1.2.5001). Continuous variables were expressed as means and standard deviations and were compared with the student’s *t* test or analysis of variance. The intra- and inter-observer reliability (absolute agreement) was assessed using intraclass correlation coefficients (ICC) with a two-way mixed effect model. Kaplan–Meier curves were used to evaluate both the subsidence over time and the implant survivorship with reoperation, complications, and stem revision as endpoints. Linear mixed models were used to evaluate changes in PROM scores over time from baseline to 1 and 2 years of follow-up (fixed effect with 3 levels), adding subject ID as a random factor.

## Results

Patients’ mean age at surgery was 64 years (SD ± 12), and the mean BMI was 29 (SD ± 4). Every patient reached a minimum follow-up of 2 years with a mean follow-up of 72.5 months (range: 29–103; SD ± 23.8). Two patients died during the follow-up due to non-surgical-related reasons.

The most common indications for stem revision were aseptic loosening in 42 cases (48%), infection in 26 (30%), instability/dislocation in 13 (15%), metal or PE wear in 5 (6%), and periprosthetic fracture in 1 case (Vancouver type A_G_ fracture) (1.1%) [32]. All removed stems were press-fit except for 3 cemented stems. A single-stage revision was performed in 64 cases (73.6%), while a two-stage revision was adopted for the rest of the 23 cases (26.4%). Intraoperatively, following the previous stem removal, Paprosky femoral bone defect type I was recorded in 53 cases (61%), and type II in 34 cases (39%).

Only 3 femoral stems were long enough to bypass the distal tip or cement mantle of the previously removed stem, while in all the other cases a de-escalation of the stem length was performed. In 5 cases, the revised stem was already a long revision stem engaging the isthmus implanted due to a previous revision THA.

The average SLCT stem size implanted was 20 (range 14–24; SD ± 2), and only 3 stems had a size inferior to 18.

During surgery, 2 cases were complicated by a trochanteric fracture and subsequently treated with cerclage wires only. In two cases a limited vertical femoral osteotomy was needed to extract the previous stem followed by metal cerclage fixation to obtain a stable trochanteric fixation. In three cases a small femoral fissure allowed the removal of the previous stem.

### Subsidence

The ICC of subsidence showed excellent absolute agreement between raters. Inter-rater reliability of the number of cm of subsidence was between 0.94 (95% CI 0.91–0.96) for 4 months, 0.96 (95% CI 0.93–0.97) for 12 months, and 0.95 (95% CI 0.91–0.97) for the 24 months post-operative rating.

The average subsidence was 2.8 mm (SD ± 3.2, range 0–15 mm) at 4 months, 3.6 mm SD ± 4.4, range 0–22.5 mm;) at 1 year, and 4.0 mm (SD ± 4.9, range 0–27.8 mm;) at 2-year follow-up. At 2 years post-operatively, 6 patients had subsidence of > 10 mm. Details of these 6 patients are reported in Table 3 [33].

**Table 3:** Details of SLCT stems with more than 10 mm of subsidence

Patient number	Time to subsidence	Possible reason of subsidence	Post-subsidence CT scan findings	Degrees of stem version at CT scan^c^	Complication related to subsidence	Reason of conus stem failure	Time to failure (months)
*1*	< 4 months	stem undersizing due to initial varus malposition	Possible change in stem version	+ 3°	None	–	–
*2*	< 4 months	stem undersizing (insufficient cortical engagement) and failure to remove cement	Possible change in stem version	− 1°	None	–	–
*3*	4–12 months	stem undersizing (insufficient cortical engagement)	No critical findings	+ 16°	None	–	–
*4*	4–12 months	intraoperative trochanteric fracture treated with cerclages which may have compromised a fully fit	No critical findings	+ 24°	None	–	–
*5*	4–12 months	stem slightly undersized due to possible failure to remove cement and/or presence of LVFO^a^ (fixed with cerclage) which may have compromised a fully fit	Cortical hypertrophy	+ 21°	Failure	Aseptic loosening	8
*6*	12–24 months	Vancouver type B1 fracture treated by ORIF^b^ with plate and cerclages	Vancouver type B1 periprosthetic fracture	+ 12°	None	–	–

Standard-length conical tapered stem

^a^Limited Vertical Femoral Osteotomy

^b^Open Reduction Internal Fixation

^c^The scans were obtained from the acetabulum to the proximal tibia with a 1.5 mm thickness using Philips Ingenuity Core 128 (Cleveland, USA). True stem version was defined as the angle between a line through the center of the neck of the femoral prosthesis and the posterior condylar line

The survival rate related to subsidence > 10 mm was 0.89 (95% CI 0.82–0.97) at 2 years.

No statistically significant relationship was found between SLCT stem size and subsidence (*p = *0.27).

### PROMs

The OHS changed from 44.5 (± 12.3) preoperatively to 25.8 (± 10.1) at the latest follow-up (*p < *0.001), while the EQ-5D improved from 0.45 (± 0.3) to 0.77 (± 0.2) (*p < *0.001).

VAS for pain during rest and activities decreased from 35.5 (± 29) and 65.9 (± 27) preoperatively to 9.8 (± 17) (*p < *0.001) and 23 (± 28) (*p < *0.001) at 2 years of follow-up, respectively.

### Complications and failures

**Fig. 1:**
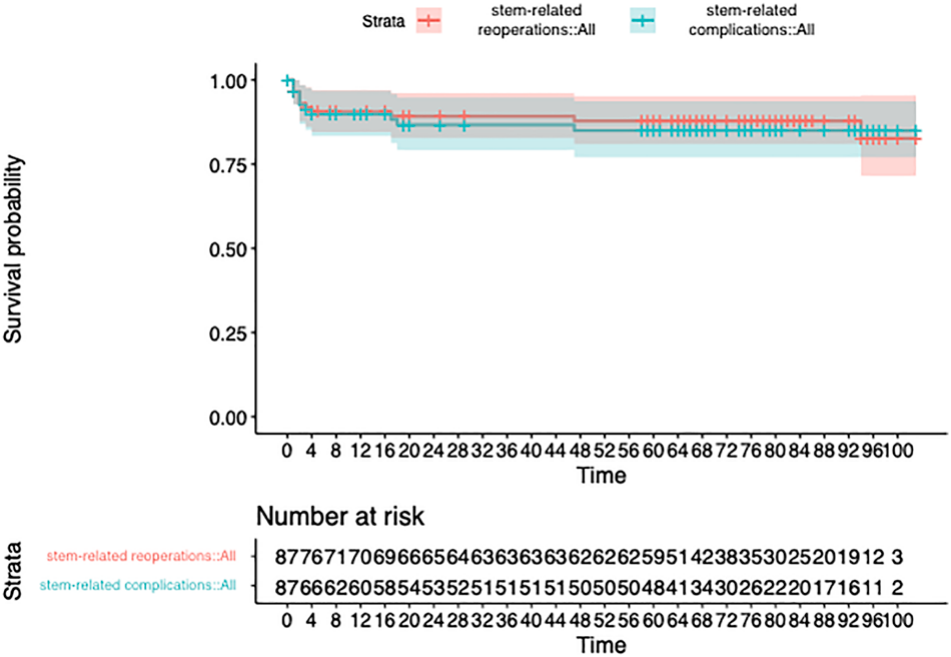
Kaplan–Meier survival curves for stem-related reoperation (in green), and stem-related complication (in blue) are presented with their 95% CI

Fifteen patients (17,2%) had at least one complication potentially related to the stem, of which 8 (9.2% of the entire population) sustained a reoperation other than stem’s re-revision while 4 (4.6% of the entire population) failed due to aseptic loosening. The survival without reoperation potentially related to the stem was 0.83 (95% CI 0.95–0.72) (Fig. 1), while the implant survival without complication potentially related to the stem was 0.85 (95% CI 0.94–0.77) (Fig. 1), with a mean time to complication of 8 months (range: 1–47 months). Details of stem-related complications and related treatments are reported in Table 4 (The overall complications, both related and not related to the stem are reported in Supplementary material as Table 4.2 in Supplementary Material). Six patients sustained a post-operative periprosthetic fracture at the tip of the prosthesis or distal to it. In one case, it was related to a significant trauma while the other 5 cases occurred without such trauma. These five patients were the only ones who had a long revision-type stem in situ at the time of revision. In two of those cases, a small femoral fissure was required to extract the previous stem. We reported 5 dislocations of which 2 occurred in patients who underwent a full THA revision (acetabular cup + femoral stem) and 3 in patients with an isolated femoral stem revision. In every case, the lateral femoral hip off-set was reduced compared to the contralateral hip (the mean hip off-set reduction was 5 mm; range 3–9 mm). None of those stems failed, but in the latter three cases, a cup revision was added to compensate for the femoral hip off-set reduction (Table 4).

**Table 4:** Stem-related complications and related treatments

Type of complication^a^	Number of patients	treatment		Number of stem failures
		Medical^b^	Surgical	
Instability/dislocation	5	–	2 = closed reduction 3 = cup revision (+ 1 abductor mechanism re-fixation with fiberwires on greater trochanter)	–
Aseptic loosening	4	–	4 = stem revision (+ 1 cerclages)	4
Periprosthetic fracture	6	1 stress fracture at the tip of the stem = no weight-bearing for 30 days	4 = ORIF^d^ with plate and cerclages (+ 1 structural graft) 1 = ORIF^d^ with cerclages only	–
TOT	15	1	14	4

^a^Values are expressed as absolute number

^b^Physical therapy and/or painkillers-analgesic drugs and/or local anaesthetic injections and/or advanced wound care

^c^Debridement, antibiotics, and implant retention

^d^Open reduction internal fixation

The overall stem survival rate was 0.93 (95% CI 0.91–0.97) at 2 years. We recorded a total of 4 mechanical aseptic loosening failures potentially related to the stem type. Only one aseptic failure was among the patients with > 10 mm of subsidence. Four other stems failed due to deep infections, therefore not for mechanical reasons related to the stem type. Details of those 8 patients are reported in Table 5.

**Table 5:** Details of failed SLCT stems

Patient number	Previous stem in place	Reason for first revision	Partial (1) or full revision (2)	Reason of conus stem failure	Time to failure (months)	∆ mm of subsidence at failure^a^	Treatment of failure
1	CLS press-fit stem removed by small femoral window/fissure	Dislocation	2	Deep infection (S. Epidermidis)	16	2.7	2-Stage revision
2	twinSys press-fit stem	Aseptic loosening	1	Deep infection (S. Epidermidis)	3	0.6	2-Stage revision
3	Zweymüller press-fit stem removed by small femoral window/fissure	Dislocation	2	Deep infection (MRSA^c^)	25	5.6	Resection arthroplasty
4	Evoke press-fit stem	Deep infection	2	Deep infection (S. Epidermidis multiresistant)	20	2.5	Resection arthroplasty
5	Exeter cemented stem removed by LVFO^d^ (fixed with cerclages)	Aseptic loosening	2	Aseptic loosening	8	10.6	1-Stage revision with diaphyseal engaging longer stem
6	CLS press-fit stem	Aseptic loosening	1	Aseptic loosening	5	4.8	1-Stage revision with diaphyseal engaging longer stem
7	Zweymüller press-fit stem	aseptic loosening	1	Aseptic loosening	29	8.2	1-Stage revision with diaphyseal engaging longer stem
8	Corail press-fit stem	Aseptic loosening	1	Aseptic loosening	47	5.5	1-Stage revision with diaphyseal engaging longer stem

Standard-length conical tapered

^a^Millimeters of difference of subsidence from the 1st day after surgery to the failure date

^c^Methicillin-resistant Staphylococcus aureus

^d^Limited vertical femoral osteotomy

## Discussion

In the present study, we demonstrated that, in the setting of revision hip arthroplasty, it is possible to address a mild–moderate femoral bone defect with a primary standard-length conical press-fit stem not engaging the femoral isthmus. Our study found an overall stem survival of 93.7% at 2 years and a survival rate without reoperation of 83% when using the SLCT stem during revision arthroplasty of Paprosky type I–II femoral bone defects. The potential use of this SLCT stem in a Paprosky type II femoral stem revision to spare bone at the femoral isthmus to address any further eventual stem revision was recently highlighted by Willems et al. [9] in a comparative study with a long monobloc revision stem. These authors concluded how uncemented primary monobloc conical femoral stems showed the same clinical result as distal fixating modular stems with fewer complications and fewer stem revisions.

Multiple studies previously showed moderate results when using different primary femoral stem designs (cemented and press-fit) in the setting of revision arthroplasty [17, 34–39]. Recently, better results have been published showing stem survival rates higher than 90% [7, 10, 17, 18, 35, 36, 40]. These studies provide promising data, but they are limited since they are short-term retrospective studies using either partially or fully coated but tapered single- or double-wedge metaphyseal filling prostheses. These primary stems achieve fixation through a tight metaphyseal fit in only one or two planes not allowing the freedom for femoral anteversion adjustments seen when using conical tapered stems.

Retrospective studies using this SLCT stem with a small population of patients have been published [40–42]. Kataham et al. performed a retrospective study using the Wagner cone in 15 revision THA surgeries and found survival of 93.3% at 33.6 months [41]. In the study of Cavagnaro et al. [40] this stem was used only in 6 patients undergoing a two-stage revision surgery due to periprosthetic joint infection. The authors reported a stem survival rate of 96.3%. Unfortunately, this survival rate includes other 63 CLS Spotorno stems; therefore, it was not possible to extrapolate the exact overall Wagner cone stem survival. Park et al. followed for an average of 10 years a total of 28 patients and did not report any Wagner cone stem failure (100% of stem survival rate) [42].

One of the endpoints of our study was to evaluate the amount of stem subsidence over time. In 6 cases we had subsidence > 10 mm and only one stem was revised due to subsidence. Considering the whole population, we did not find any difference in millimeters of subsidence between the follow-up at 12 and 24 months (average 0.4 mm of difference in subsidence). This confirms the fact that, even in the contest of sclerotic bone deriving from previous stem failures, the shape and the surface of this SLCT stem provide a stable fixation once the stem reaches its final position in the femur canal. Regarding the subsidence of a Wagner, cone stem, Cavagnaro et al. [40] reported subsidence of more than 2 mm in one out of six cases at 37.4 months, but a clear outcome was not well established. Kataham described 15 Wagner cone stems in fifteen revisions with subsidence of 2.57 mm at 33.6 months. No stems were revised in their study because of subsidence [41]. In the study of Park et al. [42], 3 of 28 (10.7%) Wagner cone stems showed subsidence of more than 5 mm that did not lead to any stem failure. In our study, the average subsidence was slightly higher (4.0 mm at 2 years of follow-up), nevertheless only one patient was re-revised due to subsidence leading to aseptic loosening.

In line with the five (17.8%) complications reported in the study by Park et al. [42], we recorded 11 complications (12.6%) potentially related to the stem. In our study, 5 complications were dislocations but none of those were treated by stem re-revision even though in every case the lateral femoral hip off-set was reduced compared to the contralateral hip. That could be related to the fact that this stem offers only limited off-set options (from 26.2 to 40 mm) that vary according to neck angles (125° and 135°) and increase as the stem size increases. This sometimes limits the possibility to re-establish native hip off-set eventually leading to dislocation.

**Fig. 2:**
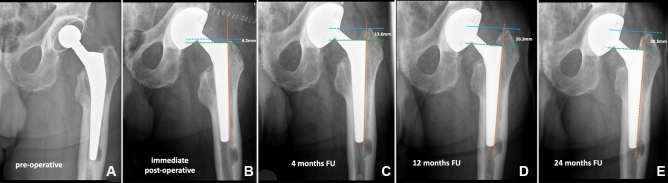
**A**–**E** show progressive subsidence of the revised stem due to undersizing (aseptic loosening). The stem size was 24, the biggest size for this specific model. **A** shows a pre-operative X-ray. The reason for the revision was aseptic loosening. **B** shows immediate post-operative X-ray control. Subsidence was 9.4 mm at 4 months of FU (**C**), 16.1 mm at 12 months of FU (**D**), and 16.3 mm at 24 months of FU (**E**). *FU* follow-up

The few reports and our study confirm that low-grade femoral defects in rTHA can possibly be treated with a primary standard-length implant [10]. In previous studies, suggested indications for using a primary stem in revision surgery were 4 cm of scratch fit in the femoral isthmus with an intramedullary canal of less than 19 mm [10], the adequate metaphyseal bone at the lesser trochanter region and 4 cm of distal fit [43], preferably a previous uncemented stem and few previous surgical interventions [17] and Paprosky I and II femoral defects [18, 35, 36]. All of our 87 patients had Paprosky type I and II femoral defects so we can agree with the fact that Paprosky I–II with enough metaphyseal bone at the region of the lesser trochanter and with 4 cm of distal fit is advisable for the use of this kind of stems [10, 43]. We cannot support the criterion that the medullary canal should not exceed 18 mm [10]. In our study, only 4 patients had a medullary femoral canal of less than 18 mm. All other patients had bigger diameters and yet showed excellent stem survival. Rather than 18 mm as a standard measurement, we recommend surgeons make sure that the maximum diameter of this prosthesis is large enough to fill up a wide empty meta-diaphyseal femoral canal (Fig. 2). Indeed, a disadvantage of using this stem is that the maximum diameter stem size is 24 mm (to fit a 24 mm medullary canal diameter), even though a custom-made implant with a bigger diameter is available on demand. It is worth noting that in our experience, we had only 4 cases (not a part of this study) where the digital pre-operative templating showed the need for *a* > 22 conus stem size and we had to switch to another longer modular revision stem due to the unsatisfactory rotational stability of the trial SLCT stem. Therefore, we recommend always to make a digital planning and keep a longer conical stem available on the day of surgery for bypassing the isthmus once the Wagner cone templating is equal to or more than 22 mm.

**Fig. 3:**
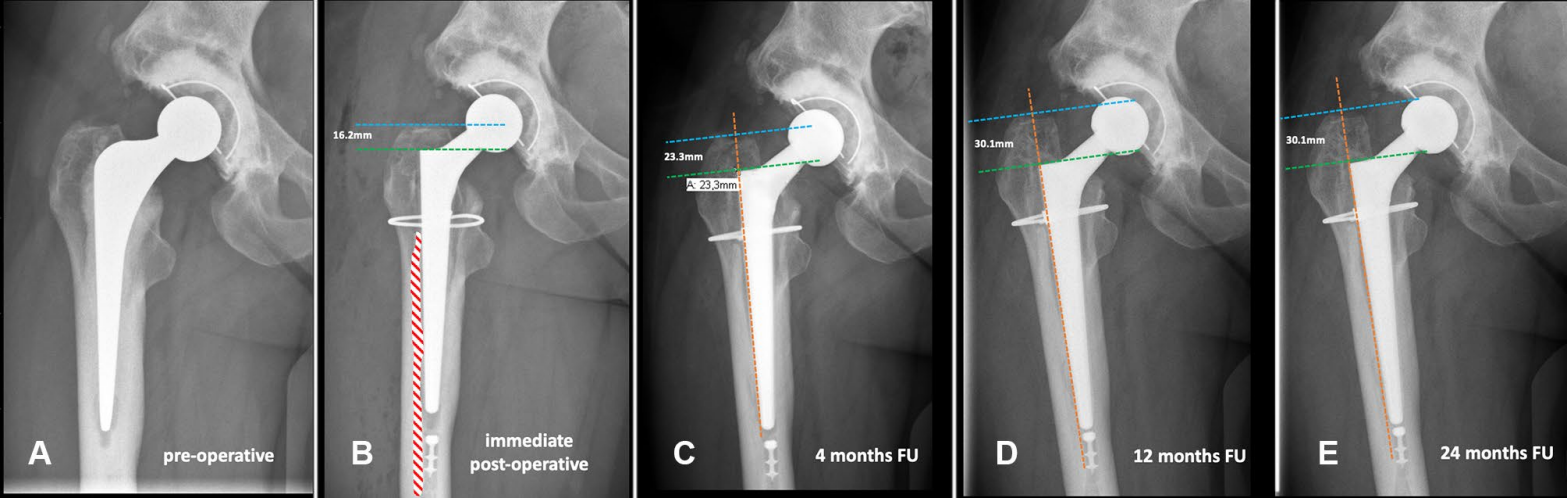
**A**–**E** show progressive subsidence of the revised stem due to insufficient cement removal (red dotted area in **B**). The reason for the revision was aseptic loosening. **A** shows a pre-operative X-ray. **B** shows immediate post-operative X-ray control. Subsidence was 7.3 mm at 4 months of FU (**C**), and 13.9 mm at 12 and 24 months of FU (**D**–**E**). *FU*  follow-up

**Fig. 4:**
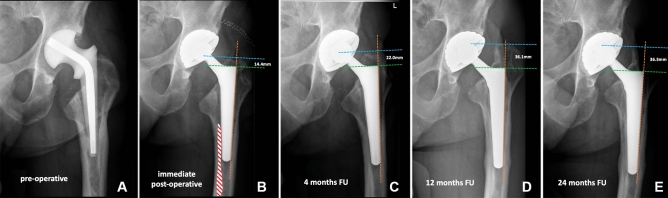
**A**–**E** show progressive subsidence of the revised stem due to insufficient cement removal (red dotted area in **B**). The initial reason for the revision was an infection of a cemented stem, and the SLCT stem was implanted after a two-stage procedure. **A** shows a pre-operative X-ray. **B** shows immediate post-operative X-ray control. Subsidence was 7.6 mm at 4 months of FU (**C**), 21.7 mm at 12 months of FU (**D**), and 21.9 mm at 24 months of FU (**E**). *SLCT stem* standard length conical tapered stem; *FU* follow-up

Additionally, our data suggest that a SLCT stem should preferably be used in cases of revising an uncemented failed stem. Indeed, 3 out of the 6 cases of subsidence > 10 mm had a previous cemented stem in place and they were the only patients in the study’s population with a cemented stem design. This could be related to the fact that it is not always easy to completely remove the cement, leading to undersizing of the conus stem, or not having proper cortical engagement (Figs. 3, 4).

**Fig. 5:**
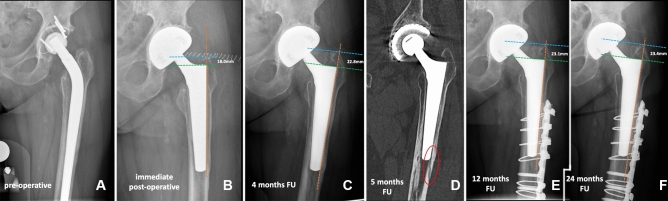
**A** shows a pre-operative X-ray of a long revision stem that failed due to aseptic loosening. **B**, **C** show a subsidence of 4.8 mm during the first 4 months after surgery. The patient referred to weight-bearing pain on the anterolateral side during the last three months. At the 5th month of FU, a CT scan was made due to the persistence of pain along the thigh (**D**), and a Vancouver type B1 fracture at the tip of the stem was detected (red ellipse). The patient was treated with ORIF with a plate, cerclages, and screws. The 12 months FU X-ray control shows complete healing of the fracture and a subsidence of 3 mm compared to the subsidence at the moment the fracture was detected (**E**). Almost no more subsidence was recorded 24 months after surgery (**F**). *FU* follow-up; *CT* computer tomography; *ORIF* open reduction internal fixation

We reported 6 post-operative periprosthetic fractures. Five occurred in patients who had a long revision-type stem in situ engaging the isthmus and implanted due to a previous THA revision. In two of those cases, a small femoral fissure was required to take out the previously fixed stem. Although the bone defect could have been classified as Paprosky type II, and the proximal femur and diaphysis had an adequate bone for implantation of a well-fixed primary short conical implant, and no subsidence > 10 mm was seen in those patients, the distal part of the femur was probably damaged and weakened due to the previous implanted long revision-type stem and/or the presence of iatrogenic femoral fissure, and most likely an insufficiency fracture occurred distal to the femoral component (Fig. 5). Therefore, we advise against the use of a primary standard-length stem if the explanted stem is a revision-type stem already engaging the isthmus or if an intraoperative femoral window/fissure was necessary to extract the previous stem with the probability of diaphyseal weakening.

Our study has some limitations. To begin with, the revisions were performed by different surgeons. Although the surgeons are extensively trained and high-volume revision hip surgeons, there is a possibility of bias. A single surgeon design would have been better to minimize confounding factors. On the other hand, having multiple surgeons in the study increases the ecological validity of the outcome. Moreover, our follow-up was relatively short even though it was comparable with other publications. Nonetheless, we do not expect that a follow-up longer than 2 years would have changed the outcomes in terms of subsidence over time. Finally, in our cohort, full and femur-only revisions were included. In some cases, the presence of a contemporary cup revision could have potentially covered any eventual complications coming from a possible stem malposition.

Based on this study, we can state that the use of this SLCT stems in the setting of femoral stem revisions with a type I–II Paprosky defect is safe and effective with good clinical results, especially in the perspective of saving the isthmus and the remaining part of the diaphysis which could be useful to have intact to face eventual future re-revisions. Despite everything, even if limited bone loss is present, there are some situations where we do not recommend its use, and these are: the previously extracted stem was cemented, the intramedullary diameter of the proximal femoral canal (above the isthmus) is > 22 mm, a femoral window/fissure was made at the apex of the previous stem to extract it, and in the setting of a re-revision (or multiple hip surgeries) where the previously extracted stem was a long revision stem already engaging the isthmus.

## Conclusions

The use of a primary SLCT stem in rTHA with Paprosky types I–II femoral bone defects demonstrated good survival with low subsidence rates during the first 2 years after surgery. Satisfactory clinical and functional outcomes were also achieved while preserving the bone stock at the femoral isthmus. Thanks to these encouraging results, we recommend that this primary conical cementless stem be considered as a potential treatment during stem revision in limited femoral bone loss.

## Supplementary Information

Below is the link to the electronic supplementary material.

Supplementary file1 (DOCX 22 kb)

Supplementary file2 (DOCX 15 kb)

## Data Availability

The authors confirm that the data supporting the findings of this study are available within the article [and/or] its supplementary materials.
