# Supplementary material for: The use of a standard-length conical tapered stem in hip revision arthroplasty to address Paprosky type I–II femoral defects: a prospective study of 87 patients
Source: Arch Orthop Trauma Surg. 2023 Feb 20;143(9):5945–55. doi: 10.1007/s00402-023-04797-y (PMC10449674; doi:10.1007/s00402-023-04797-y)
Supplement: Supplementary file 1 — Supplementary file1 (DOCX 22 kb) [file 402_2023_4797_MOESM1_ESM.docx]

**Table 4.2** Overall complications and related treatments

| **Type of Complication^§^:** | **number of patients** | **treatment** | | **number of stem failures** |
| --- | --- | --- | --- | --- |
|  |  | ***medical ^£^*** | **surgical** |  |
| - soft tissue related pain | 8 | 3  3 = psoas injection | 1 = trochanteric plate removal  1 = trochanteric cerclage removal | / |
| - deep infection | 9 |  | 5 = DAIR^#^  4 = two stage revision | 4 |
| - instability/dislocation | 5 | / | 2 = closed reduction  3 = cup revision (+ 1 abductor mechanism re-fixation with fiberwires on greater trochanter) | / |
| - wound problems (leakage/dehiscence) | 1 | 1 |  |  |
| - aseptic loosening | 4 | / | 4 = stem revision (+ 1 cerclages) | 4 |
| - periprosthetic fracture | 6 | 1 stress fracture at the tip of the stem = no weight bearing for 30days | 4 = ORIF^&^ with plate and cerclages (+ 1 structural graft)  1 = ORIF^&^ with cerclages only | / |
| - intraoperative positive culture | 5 | 5 = antibiotics for 12 weeks | / | / |
| TOT. | 37 | 12 | 25 | 8 |

^§^ Values are expressed as absolute number

^£^ Physical therapy and/or painkillers-analgesic drugs and/or local anaesthetic injections and/or advanced wound care

^#^ Debridement, Antibiotics, and Implant Retention

^&^ Open Reduction Internal Fixation
