# Supplementary material for: The use of a standard-length conical tapered stem in hip revision arthroplasty to address Paprosky type I–II femoral defects: a prospective study of 87 patients
Source: Arch Orthop Trauma Surg. 2023 Feb 20;143(9):5945–55. doi: 10.1007/s00402-023-04797-y (PMC10449674; doi:10.1007/s00402-023-04797-y)
Supplement: Supplementary file 2 — Supplementary file2 (DOCX 15 kb) [file 402_2023_4797_MOESM2_ESM.docx]

| Model | Brand | Number |
| --- | --- | --- |
| Secure-Fit Advanced | Stryker Orthopedics, Nahawa NJ, USA | 2 |
| Accolade II |  | 7 |
| Exeter * |  | 2 |
| Restoration Modular (body size 70mm, conical distal stem length 155mm) |  | 1 |
| SP-CL | Waldemar Link GmbH & Co, Germany | 4 |
| SP-II * |  | 1 |
| MP (proximal segment 35mm, stem length 160mm) |  | 1 |
| PolarStem | Smith & Nephew, Memphis, TN, US | 6 |
| Profemur-Z | MicroPort Orthopedics Inc., USA | 2 |
| Corail | DePuy, Raynham, MA, USA | 9 |
| Trilock |  | 3 |
| S-ROM (N standard 130mm) |  | 3 |
| Taperloc standard | Zimmer-Biomet, Warsaw, Indiana, USA | 4 |
| Taperloc Microplasty |  | 1 |
| Arcos (vertical body height 60mm, distal stem length 115mm) |  | 1 |
| Fitmore B |  | 1 |
| CLS |  | 11 |
| Zweymuller |  | 18 |
| Avenir |  | 8 |
| Wagner SL (190mm – current version) |  | 1 |
| Wagner SL (265mm – older version) | Sulzer, Winterthur, Switzerland | 1 |

* cemented
